# A General Practitioner's Views about Locums

**Published:** 1908-08-15

**Authors:** 


### The General Practitioner's Column.

[Contributions to this Column are invited, and if accepted will be paid for.]

### A GENERAL PRACTITIONER'S VIEWS ABOUT LOCUMS.

When I read during last summer in the columns of The Hospital a page dealing with the relations of a locum-tenens to his principal, and an invitation to practitioners to give their point of view, I felt, with some self-satisfaction, that my own experiences with my deputies had been fairly unexciting. Since then I have heard from some of my professional neighbours strange tales, and so I feel justified in giving my views as to the best ways of escaping unpleasantness with one's holiday substitute.

First, let me lay down a few elementary principles. I advise any medical man who requires a locum never, except under compulsion, to take one unless he can get some reliable information about the latter's qualifications from a friend. By qualifications I do not mean the exact letters he is entitled to write after his name, but the possession of energy, tact and address. I would rather have a man who has the knack of getting on with patients of all classes, no matter how poor his academical distinctions, than a much-examined man who lacks that quality. Get, if it is possible, a man who has given satisfaction to a neighbour, rather than a total stranger, however good his testimonials. Next it will always be found economical to give a good salary. This pays well in every way, for one's quondam locum, or assistant, when settled in practice and no longer available, can sometimes recommend a friend; and if he knows that a liberal salary is to be given he will take care not to send any but a good man: moreover, he knows the requirements of the particular practice, and this is a great advantage.

I note the would-be locum-tenens is advised to apply to the Dean of his school, through whom he may often get employment. I think this method of obtaining a deputy may be a good one for the practitioner, especially if he and the Dean are personal friends of student days. It always strikes me, however, that if one does not know the Dean one is less likely to get the pick of his promising young men, because naturally his friends get best served. Medical agents of approved standing are often useful. They soon find out the quality of the men on their books, and when it is plain to them that none but a good man will satisfy a client, in their own interests they can be trusted to do their best. Those who have bought a practice through an agent often apply to him regularly each year for a locum tenens; this plan answers very well in my experience.

Some of my fellow-practitioners have tried communicating with gentlemen who advertise their services in the medical papers, and they seem to think that, as by all other methods of getting a locum, they draw both blanks and prizes: the risk of accepting an unknown man in this species of lottery has so far deterred me from adopting the plan. The reverse way, that of advertising one's want of a locum for a certain date, seems for some reason very little in favour. Yet a practitioner of great shrewdness and long-standing tells me that he does it sometimes, and that he can always select a good man by a careful examination of the answers he receives. After all, if a doctor who has been twenty-five years in practice is not a judge of men, he has probably mistaken his vocation, which is certainly not the case with the gentleman in question.

I think it is always worth while to give the locum a good introduction to one's patients. Some men rush off by the next train as soon as he enters the house, and many make only one casual round with him to some of the more acute cases: I make it a rule not to leave my practice until thirty-six or forty-eight hours after my locum arrives, and to introduce him to every patient actually under treatment. The prejudices and peculiarities of patients are trying enough to oneself, but to a locum, brought suddenly into contact with an exaggerated form of them, they must be absolutely appalling.

Your locum-tenens contributor explained how useful it is to a man contemplating practice to do first a locum-tenency. That advice is good, not only for the reasons put forward, but also because he may thus obtain an insight into the difficulties of his deputies in after years, and so get the best work out of them with the least worry to himself. The knowledge of how to conduct a practice can be as well or better attained by spending a year or so as an assistant which has always seemed to me a most useful thing for a young man without much capital to do: he may not learn so much operative surgery, or such recent and ephemeral fads of medical diagnosis as in a resident hospital appointment, but he learns more dispensing and practical therapeutics, which will be useful if his future is to be spent in a middle-class practice in a country town.

Now for one or two actual experiences. Not every man who offers himself as a locum-tenens is really a qualified medical man. It seems hard to believe, but it is, nevertheless, a fact that many medical men accept a locum's account of himself without any attempt to verify it by reference to the Medical Directory or the Register. That precaution is one that most of us do take, but two cases that have come to my personal knowledge show that it is not really enough when dealing with a total stranger. One is that of a man who was imprisoned in 1906 for falsely assuming the name and qualifications of a registered practitioner, who now lives on the other side of the Atlantic Ocean. He thus obtained a locum-tenency in a village known to me, and his employer had the mortification of having a testimonial (written by him on his return) read in court when the rogue was tried for, and convicted of, fraud over the sale of a small practice in another part of the country. Fortunately that practitioner's patients did not get to hear that they had been committed to the care of an impostor.

In another case, a man who had been an unqualified assistant, until the action of the Medical Council a few years back made that position no longer tenable, successfully deceived several medical men by assuming the name of a late employer, then deceased. If the practitioner called attention to the fact that his name was not in the Directory, he replied that it had been removed owing to his negligence in not answering circulars; but, he would add, you will find it all right in a copy three or four years' old, which was, of course, the case. He thus deceived an acquaintance of mine; it is only fair to say that this man had a very sound knowledge of all simple ailments, and enough common sense to seek a second opinion on most others, so no great harm was done.

Locums who have been phenomenally stupid, careless, or unsociable, I have occasionally met with, and have often wondered what becomes of them in the profession ultimately; perhaps they are buying experience (at their employer's expense), which transforms them into successful men. Unscrupulous or vicious ones are very rare, though some queer stories are told now and then. Once a gentleman took charge of a large working-class practice, whose owner had a big reputation locally for midwifery, and caused some consternation by an absolute refusal to budge from bed between midnight and 8 a.m. He certainly had to work pretty hard during the day, but he had fair warning of what he might expect and liberal treatment in every way. The locum who carries on half a dozen flirtations with the daughters of one's best patients, or "rubs the wrong way" the vicar of the parish when visiting the sick, or forgets to make up Mrs. Blank's digitalis mixture, on which she has sustained life for two years, because he is in a hurry to reach the links in time for a round, I suppose everyone comes across at some time or other. He who entertains doubts on the diagnosis of three-fourths of one's cases, and expresses them, is bad enough; but worse still, according to my friends, are those who offend patients, cause the cook to give notice and the coachman to take to drink, and then feel aggrieved at not being allowed to retain notification fees. Occasionally it is the locum who displays alcoholic tendencies, but the surest way of avoiding this kind of trouble is never to engage a stranger unless recommended by somebody one knows.

On the question of agreements there is much laxity in the profession: when one secures a locum through an agent, one has a signed form setting forth the exact conditions of the engagement and prohibitory clauses as to commencing practice in the neighbourhood within a certain length of time. This is, of course, the business-like and proper way, but very often there is no understanding other than a purely verbal one, which, though I plead guilty to the practice myself, is certainly a foolish thing to do. Suppose one broke a leg on the return journey to one's home; if the locum-tenens was doing well one would feel very well pleased if he consented to continue acting. But one would be rather annoyed if, at the end of three months, he put up a plate in the next street, and attracted a certain number of the patients. I do not think I have had a locum who would do that, but one never quite knows what may happen.

Finally may I assure any members of the profession who now or subsequently may undertake the duties of a locum-tenens, that medical men in practice appreciate their difficulties, and are always willing and ready to help or advise their neighbour's deputy in any trouble or emergency.

